# Cellular senescence: from homeostasis to pathological implications and therapeutic strategies

**DOI:** 10.3389/fimmu.2025.1534263

**Published:** 2025-02-03

**Authors:** Chunhong Li, Yixiao Yuan, YingDong Jia, Qiang Zhou, Qiang Wang, Xiulin Jiang

**Affiliations:** ^1^ Department of Oncology, Suining Central Hospital, Suining, Sichuan, China; ^2^ Department of Medicine, Health Cancer Center, University of Florida, Gainesville, FL, United States; ^3^ Gastrointestinal Surgical Unit, Suining Central Hospital, Suining, Sichuan, China

**Keywords:** cellular senescence, cancer, m6A RNA methylation, aging, longevity, inflammation

## Abstract

Cellular aging is a multifactorial and intricately regulated physiological process with profound implications. The interaction between cellular senescence and cancer is complex and multifaceted, senescence can both promote and inhibit tumor progression through various mechanisms. M6A methylation modification regulates the aging process of cells and tissues by modulating senescence-related genes. In this review, we comprehensively discuss the characteristics of cellular senescence, the signaling pathways regulating senescence, the biomarkers of senescence, and the mechanisms of anti-senescence drugs. Notably, this review also delves into the complex interactions between senescence and cancer, emphasizing the dual role of the senescent microenvironment in tumor initiation, progression, and treatment. Finally, we thoroughly explore the function and mechanism of m6A methylation modification in cellular senescence, revealing its critical role in regulating gene expression and maintaining cellular homeostasis. In conclusion, this review provides a comprehensive perspective on the molecular mechanisms and biological significance of cellular senescence and offers new insights for the development of anti-senescence strategies.

## Introduction

### Mechanisms of cellular senescence

Cellular senescence is a complex and multifaceted biological process characterized by a stable arrest of the cell cycle in response to various stressors, such as DNA damage, oxidative stress, and oncogene activation (1). Although senescent cells no longer proliferate, they remain metabolically active and exhibit distinct phenotypic changes, including the secretion of pro-inflammatory factors, collectively termed the senescence-associated secretory phenotype (SASP) (2, 3). Senescence plays dual roles in physiological and pathological contexts: it is essential for processes like tissue remodeling, wound healing, and tumor suppression, yet its accumulation contributes to aging, chronic inflammation, and the progression of age-related diseases, including cancer and neurodegenerative disorders (4). Understanding the mechanisms underlying cellular senescence is crucial for developing therapeutic strategies to harness its beneficial aspects while mitigating its detrimental effects.

### Functions of m6A modification

m6A (N6-methyladenosine) RNA modification has emerged as a key regulator of cellular processes, including senescence. m6A is the most prevalent internal modification in eukaryotic mRNA and is dynamically regulated by “writers” (methyltransferases, such as METTL3 and METTL14), “erasers” (demethylases, such as FTO and ALKBH5), and “readers” (m6A-binding proteins, such as YTHDF1 and YTHDC1) (5–9). By modulating RNA stability, splicing, translation, and decay, m6A modifications influence a wide array of biological functions, including cell proliferation, differentiation, and stress responses (10–13). Recent studies have highlighted the role of m6A in regulating the pathways associated with cellular senescence, including p53, NF-κB, and SASP components. However, the intricate interplay between m6A modifications and senescence remains incompletely understood, warranting further exploration.

This review aims to provide a comprehensive overview of the mechanisms and implications of cellular senescence, with a particular focus on the emerging role of m6A RNA modifications. By synthesizing current knowledge, this review highlights the dual roles of senescence in homeostasis and pathology, elucidates the regulatory functions of m6A modifications, and discusses the potential of targeting m6A for therapeutic intervention. Additionally, this review identifies key challenges and future directions in the field, providing valuable insights for researchers and clinicians alike. Through an interdisciplinary approach, this work seeks to advance our understanding of senescence and its regulation by m6A, ultimately contributing to the development of novel strategies for age-related diseases and cancer therapy.

### The dynamic regulation of m6A modification

m6A modification is the most prevalent internal modification found in eukaryotic mRNA. It plays a crucial role in regulating various aspects of RNA metabolism, including stability, splicing, translation, and decay. The dynamic nature of m6A modification allows cells to fine-tune gene expression in response to developmental cues, environmental stresses, and disease states, making it a key player in cellular processes such as differentiation, cell cycle progression, and response to DNA damage. The process of m6A modification is highly regulated by a set of specific enzymes, including methyltransferases (“writers”), demethylases (“erasers”), and m6A-binding proteins (“readers”).

### Methyltransferases (Writers)

In the m6A writer complex, several proteins have been identified, including METTL3, METTL14, METTL16, WTAP, RBM15, KIAA1429, and ZC3H13 (14–19). The m6A modification is catalyzed by a complex of methyltransferases, primarily composed of METTL3 and METTL14. METTL3, the catalytic core of the complex, adds a methyl group to the nitrogen-6 position of adenosine residues in RNA (20). METTL14 functions in concert with METTL3 to stabilize the mRNA and provide specificity to the methylation process (21). In addition to METTL3 and METTL14, other cofactors such as WTAP (Wilms tumor 1-associating protein) and KIAA1429 (VIRMA) contribute to the proper functioning of the methyltransferase complex, ensuring that the m6A marks are deposited at specific regions within the RNA molecule (22, 23). These “writers” play a central role in modulating RNA stability and translation, impacting gene expression on both a short-term and long-term basis (24).

### Demethylases (Erasers)

The erasure of m6A modifications is carried out by specific demethylases, with the most well-characterized being FTO (fat mass and obesity-associated protein) and ALKBH5 (alkylated DNA base repair homolog 5) (25–28). These enzymes remove the methyl group from the adenosine, reversing the m6A modification (29). FTO and ALKBH5 play essential roles in regulating RNA stability and are implicated in various biological processes, including energy homeostasis, neuronal function, and response to stress (30)s. The activity of demethylases ensures that m6A modifications are reversible and allow cells to dynamically control the presence or absence of m6A marks on RNA, which is critical for maintaining RNA homeostasis and regulating gene expression in response to cellular needs (31). Additionally, a concurrent study revealed that the acetylation modification on K235 of ALKBH5, along with its regulatory subunit PSPC1, jointly determines the m6A demethylase activity and oncogenic function of ALKBH5. The K235 acetylation of ALKBH5 enhances its binding recognition to substrate RNA m6A (32), thus augmenting ALKBH5 removal of m6A modifications on RNA.

### M6A reader proteins

m6A can be recognized by various m6A-binding proteins that play multiple roles in regulating gene expression. These effector proteins are referred to as m6A ‘readers’. Major m6A readers include the YTH domain-containing protein family, such as YTHDF1, YTHDF2, YTHDF3, YTHDC1, and YTHDC2 (10, 33–36). The effects of m6A modification are primarily mediated by m6A-binding proteins, or “readers,” which recognize and bind to the modified adenosine residues in mRNA. These reader proteins include YTHDF1, YTHDF2, YTHDC1, and IGF2BP1, among others. Each reader has a unique role in modulating RNA fate; for example, YTHDF1 enhances mRNA translation, while YTHDF2 promotes mRNA degradation. IGF2BP1, another critical reader, stabilizes m6A-modified mRNAs by protecting them from degradation. These reader proteins essentially translate the m6A modification into functional outcomes by either facilitating RNA processing, enhancing translation, or promoting decay, depending on the cellular context and the specific reader protein involved. Together, the coordinated action of these “writers,” “erasers,” and “readers” orchestrates the dynamic regulation of m6A modifications in RNA. Their precise control over mRNA fate is crucial for maintaining cellular homeostasis and regulating gene expression across various biological processes, including development, stress responses, and disease progression. As such, the m6A modification system is a vital component of post-transcriptional regulation, influencing a wide range of cellular functions.

### Cellular senescence

Cellular Senescence can promote tissue remodeling but may also lead to reduced regenerative potential and tissue function, consequently inducing inflammation and tumorigenesis in aging organisms (37, 38). Therefore, identifying, characterizing, and pharmacologically eliminating senescent cells is a key focus in current aging research. However, the nonspecific nature of senescence markers and the diverse senescence processes present major challenges in aging studies. The characteristics of senescence include a gradual loss of physiological integrity, impaired functionality, and increased susceptibility to death.

### Characteristics of cellular senescence

**Figure 1:**
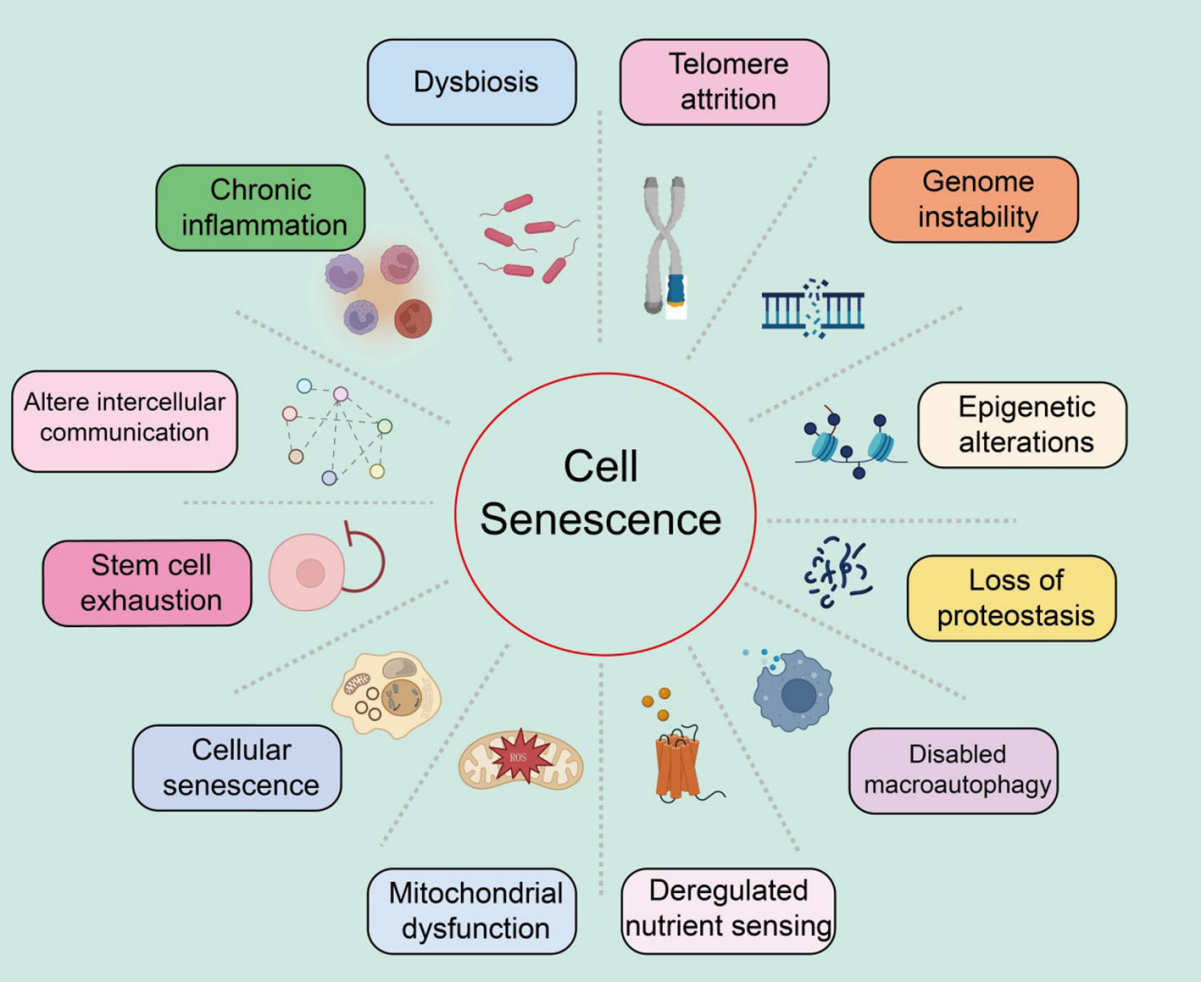
Summary of the characteristics of cellular senescence, with various features as shown in the text above.

We systematically summarized the characteristics of cellular senescence, as well as the distinct features of each characteristic (**Figure 1**).

### Genomic instability

Genomic instability refers to the process where the integrity and stability of the genome are compromised by various exogenous factors (such as chemical, physical, and biological agents) and endogenous factors (such as chromosomal segregation defects) (39, 40). These damages can induce a wide range of genetic variations, including point mutations and deletions. These molecular changes and the resulting genomic rearrangements can lead to both normal and pathological aging (5).

### Telomere attrition

Telomere attrition refers to the damage to the DNA at the ends of chromosomes (known as telomeres), leading to cellular senescence and age-related diseases (41–43). The replicative DNA polymerase (telomerase) cannot fully replicate the DNA at the telomeric regions of eukaryotic chromosomes (44). Consequently, after several rounds of cell division, telomeres significantly shorten, resulting in genomic instability and ultimately triggering apoptosis or senescence. Telomerase, an active ribonucleoprotein, extends telomeres through its reverse transcriptase activity to maintain sufficient telomere length (45). Studies have shown that telomere attrition inhibits the tumorigenic process by limiting the replicative potential of tumor cells, contrary to the genomic instability that promotes tumorigenesis (46). Furthermore, telomerase deficiency is linked to the early onset of several diseases (47). Shelterin proteins can block DNA damage and regulate telomere length, and can accelerate tissue aging if their function is lost, even if telomere length is normal (48). Significant shortening or extension of telomere length can notably affect the lifespan of mice while reactivating the telomerase gene can reverse the early aging phenotype in telomerase-deficient mice (49). Additionally, pharmacological activation of telomerase or systemic viral transduction can delay normal aging processes in mice, with telomere hyper-lengthening in mice exhibiting extended lifespan and improved metabolic features (50).

### Epigenetic alterations

Ample evidence suggests that epigenetic changes contribute to degradation pathways to aging. These regulatory modifications, which typically affect gene expression and other cellular processes reversibly, can promote the development and progression of various diseases, such as cancer, neurodegeneration, metabolic syndromes, and bone diseases (51, 52). These changes impact gene expression and other crucial cellular processes, playing significant roles in aging and age-related human pathologies, including cancer.

### Loss of proteostasis

All cells need to maintain the stability and functionality of their proteome through a series of quality control mechanisms (53). The mechanisms that maintain proteostasis include the proper folding of proteins and their degradation via proteasomes or lysosomes (54). Chronic accumulation of unfolded, misfolded, or aggregated proteins often form intracellular inclusions or extracellular amyloid plaques, promoting the development of certain age-related diseases (55). Various factors, such as oxidative stress, genetic mutations, infections, and a lack of chaperone proteins, can induce endoplasmic reticulum stress, leading to the accumulation and aggregation of proteins (56). To cope with this stress, the endoplasmic reticulum initiates the unfolded protein response (UPR), which reduces protein synthesis, expands the endoplasmic reticulum, and expels misfolded proteins (57). The increase in UPR in aging cells may be a response to the increased protein synthesis required for the SASP.

### Dysfunction of macroautophagy

Macroautophagy involves the sequestration of cytoplasmic material within double-membraned vesicles, which then fuse with lysosomes to digest their contents (58). Thus, autophagy not only plays a role in proteostasis but also affects the degradation of non-protein macromolecules, entire organelles, and invading pathogens (59). Age-related declines in autophagy are a key mechanism for the reduced renewal of organelles, validating autophagy as a hallmark of aging (60). It should be noted that the genes and proteins involved in the autophagy process also play roles in other degradation pathways, the exosomal form of intracellular waste disposal, which is subsequently cleared by macrophages (61).

### Deregulated nutrient sensing

The nutrient-sensing network is highly conserved through evolution, including extracellular ligands such as insulin and insulin-like growth factors, interacting receptor tyrosine kinases, intracellular signaling cascades like the PI3K-AKT pathway, and transcription factors such as FOXO and E26, which transcribe genes involved in various cellular processes (44, 46, 62). Under conditions of nutrient abundance and low stress, cells respond by activating anabolic processes; in contrast, under nutrient scarcity and increased stress, cells induce defensive pathways (48). Studies have shown that variations in the FOXO3 transcription factor and genes encoding components of the nutrient-sensing network are associated with human longevity (63). In human cells, epigenetic age is also related to nutrient sensing. While this signaling network promotes beneficial anabolic processes in youth, it may contribute to aging in adulthood.

### Mitochondrial dysfunction

Mitochondria are not only the energy source of cells but also potential triggers of inflammation. When ROS or mitochondrial DNA leak from the organelles, they respectively activate inflammasomes or cytoplasmic DNA sensors, leading to cell death (64, 65). With age, mitochondrial dysfunction increases, mainly manifesting as the accumulation of mitochondrial DNA mutations, instability of respiratory chain complexes, proteostasis defects, reduced organelle renewal, and changes in mitochondrial dynamics (66, 67). These factors increase ROS production and may lead to high permeability of the mitochondrial membrane, triggering inflammation and cell death. Thus, mitochondrial function is crucial for maintaining health.

### Stem cell exhaustion

Studies have reported that in the skin epidermis, characterized by high turnover and susceptibility to damage, multiple stem cell niches exist, particularly those associated with hair follicles, capable of renewal and repair (56, 68). Indeed, tissue repair largely depends on injury-induced dedifferentiation and plasticity of cells. For example, in the intestines, brain, and lungs, injury induces dedifferentiation of non-stem cells, reactivating normally silent embryonic and stem-like transcription programs to acquire the plasticity required for tissue repair (69, 70). Injury-induced plasticity may be more related to aging. Stem cells and progenitor cells, like cells without stem cell potential, exhibit similar characteristics of senescence (71). Aging is associated with gradual changes in intercellular communication, impairing homeostasis, and hormonal regulation.

### Altered intercellular communication

Aging is closely related to gradual changes in intercellular communication, which impair homeostasis and hormonal regulation (72). Increased inflammatory responses, reduced immune surveillance of pathogens and precancerous cells, and altered bidirectional communication between the human genome and microbiome eventually lead to dysbiosis (73). Many studies focus on identifying bloodborne systemic factors with pro-aging or life-extending properties, the role of different intercellular communication systems, and assessing the functional relevance of extracellular matrix destruction during aging.

### Chronic inflammation

Inflammation increases during aging, typically accompanying systemic manifestations and local pathological phenotypes, including atherosclerosis (74). Consequently, circulating levels of inflammatory cytokines and biomarkers increase with age (75). Elevated plasma IL-6 is a predictive biomarker for all-cause mortality in the elderly (76). As inflammation increases, myeloid cells and lymphocytes in the tissues and blood of patients and mice. The inflammatory response increases during aging, creating a condition known as “inflammaging,” which leads to many age-related diseases, such as osteoarthritis, atherosclerosis, sarcopenia, and neuroinflammation (77, 78). Similarly, inflammation is a contributory feature of cancer.

### Dysbiosis

In recent years, the gut microbiome has emerged as a key factor in various physiological processes (79). The gut microbiota also signals to the peripheral and central nervous systems as well as other distant organs, playing a crucial role in the overall maintenance of host health (80). Disruption of this bacteria-host bidirectional communication can lead to cancer. Advances in this field have sparked great interest among researchers in exploring changes in the gut microbiota during aging.

## Summary of senescence markers in different tissues

### Cardiovascular system

**Figure 2:**
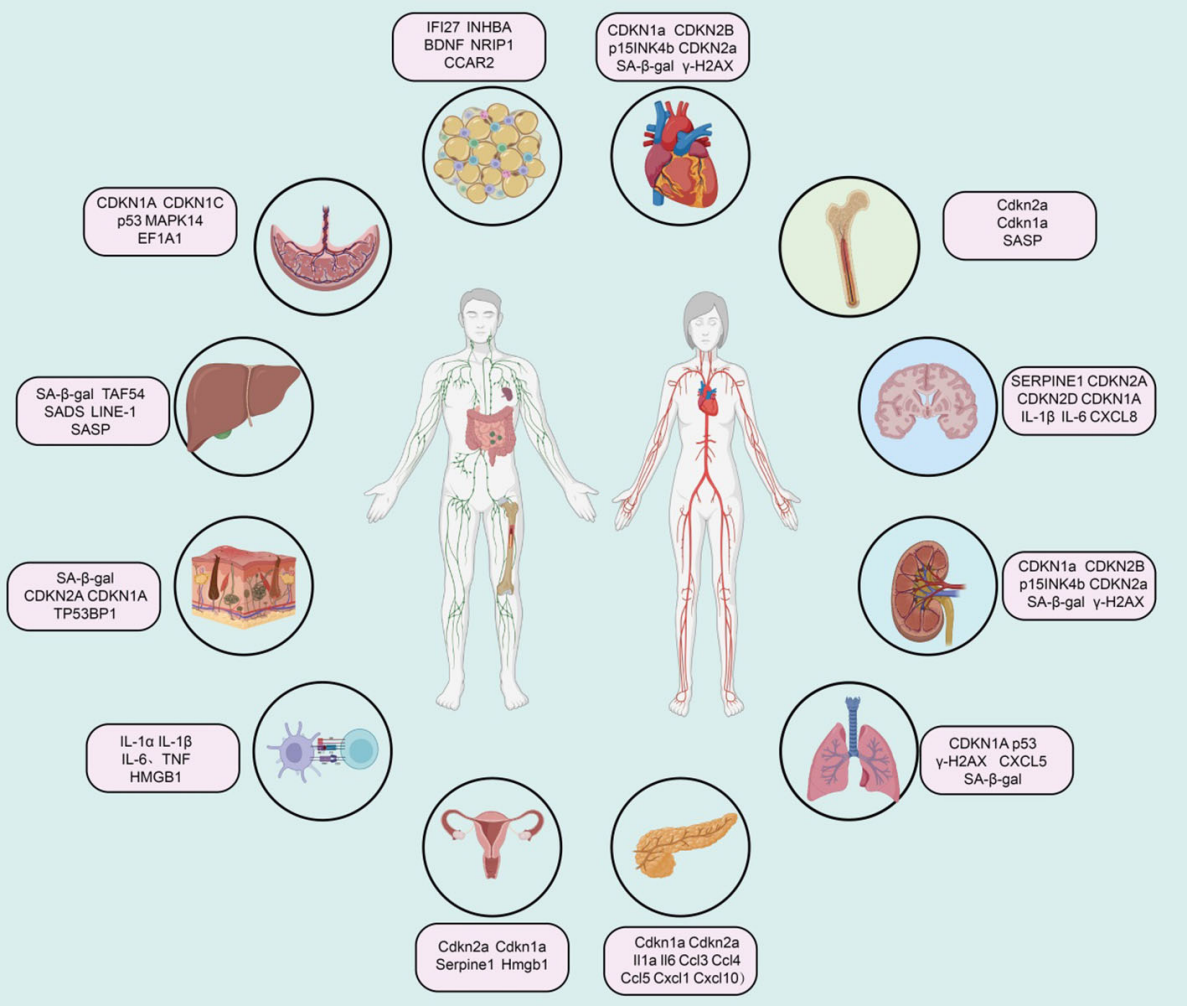
Summary of senescence marker genes in different tissues.

Extensive research indicates that various cell lineages within the cardiovascular system, including cardiomyocytes, cardiac mesenchymal cells, cardiac fibroblasts, and cardiac progenitor cells, accumulate senescence-associated markers in the context of aging and cardiovascular disease (81, 82). During normal aging, murine cardiomyocytes accumulate markers such as telomere-associated foci (TAF), cyclin-dependent kinase inhibitors (CDKN1a, CDKN2B, i.e., p15INK4b, and CDKN2a), SA-β-gal activity, senescence-associated DNA segments (SADS), and pro-fibrotic SASP (83). Vascular aging manifests as arterial thickening, stiffness, and endothelial dysfunction (**Figure 2**).

### Immune system

Immunosenescence increases susceptibility to infectious diseases and reduces vaccine efficacy. This phenotype may be related to the senescence of immune cells, particularly T cells. Studies indicate that immune cells, including monocytes and tissue-resident macrophages, are major sources of SASP factors (e.g., IL-1α, IL-1β, IL-6, TNF, and HMGB1) (84). Recent studies on platelets in elderly individuals have found elevated levels of SELP (CD62P), CD40LG, and CD63, indicating increased age-related platelet activation (85). A high SA-β-gal activity subset of CD8A+ T cells (also showing telomere dysfunction and Cdkn2a-mediated senescence characteristics) and CD8A+ effector memory T cells exhibit SASP regulated by p38 MAPK (86). Notably, peripheral mononuclear cells expressing CDKN2A seem unable to survive cryopreservation, so quantifying CDKN2A mRNA levels in peripheral T cells primarily involves freshly isolated CD3+ cells to ensure the characterization of senescent subpopulations (87). Recent reports suggest that circulating senescent myeloid cells may drive neurodegeneration and brain inflammation (88).

### Bone marrow

In a mouse model of acute myeloid leukemia, qPCR analysis of bone marrow cells revealed elevated expression of aging markers Cdkn2a, Cdkn1a, and SASP factors, while the expression of Lmnb1 was reduced (89). In radiation-induced senescence models, there was a significant increase in the proportion of cells positive for SA-β-gal activity and markers of Cdkn2a, Cdkn1a, and various SASP factors (90). Similarly, bone marrow samples from elderly individuals also exhibited markers of bone marrow aging. qPCR analysis of bone and bone marrow tissue biopsies showed increased levels of CDKN2A and CDKN1A, with SASP factors rising with age (91). Single-cell transcriptomic studies have also shown a significant increase in the expression of CDKN1A, TGFB1, and SASP factors with aging (92).

### Central nervous system

In post-mitotic cells and progenitor cells of the central nervous system in both humans and mice, aging and disease contexts are associated with overexpression of aging-related cell cycle regulators (e.g., CDKN2A, TP53, CDKN1A) and SASP factors (93). Elevated SA-β-gal activity, increased expression of apoptotic (Bcl2) and DNA damage (γ-H2AX and phosphorylated p38 MAPK) regulatory factors, and downregulation of LMNB1 are additional features of neuronal and glial cell aging or neurodegenerative pathology (94). Single-cell sequencing of the brains of Alzheimer’s disease patients shows that aging markers in excitatory neurons are consistent with Tau pathology, and CDKN2D has been identified as a prominent aging marker for Alzheimer’s disease (95). In Parkinson’s disease patients, SATB1 (a CDKN1A inhibitor) levels are reduced in the substantia nigra, while CDKN2A, MMP3, IL-6, IL-1α, and CXCL8 levels are elevated, indicating an increased aging burden (96). Finally, oxidative stress (e.g., radiation), metabolic stress, and inflammatory stress (e.g., obesity and alcohol toxicity) in mouse models summarize age-related and disease-related aging features in the mouse brain (97).

### Adipose tissue

The accumulation of senescent cells in adipose tissue is associated with tissue dysfunction in aging and age-related diseases (98). Senescent cells in human and mouse adipose tissue exhibit high expression of CDKN2A and CDKN1A, elevated SA-β-gal activity, DNA damage (γ-H2AX) and cell proliferation arrest (MKI67), and increased expression of SASP factors (99). A single-cell transcriptomic study identified a high senescence-like cell population with Cdkn1 in adipose tissue-derived mesenchymal stem cells from aged mice, accompanied by upregulation of Il6, Il15, Vegfa, Cxcl2, Ccl2, and Cxcl1 (100). Mature human adipocytes undergo senescence under obesity and hyperinsulinemia stimulation, exhibiting premature aging transcriptomic and secretory characteristics, including upregulation of CDKN2A, CDKN1A, and CCND1 gene expression, and downregulation of HMGB1 and HMGB2 gene expression (101). Additionally, increased secretion of SASP proteins CXCL8, SERPINE1, CXCL2, and MMP14 is observed. Moreover, under conditions like aging, certain markers in adipose tissue show a positive correlation with aging, including IFI27, and CCAR2 (102).

### Kidneys

Various types of kidney cells in humans and mice exhibit aging phenomena in the context of healthy aging and kidney diseases (103). Senescent glomerular endothelial cells overexpress CDKN1A, TP53BP1, γ-H2AX, and SERPINE1 (104). Overexpression of SERPINE1 leads to podocyte detachment and apoptosis, resulting in glomerulosclerosis (105). In the kidneys of aged mice, podocytes also overexpress various aging markers and SASP factors. In early diabetic kidney damage, SERPINE1 drives glomerulosclerosis (106). Most human kidney diseases are related to accelerated cellular senescence, particularly with overexpression of CDKN2A in TECs. In glomerular diseases, nuclear CDKN2A levels are elevated in glomerular and interstitial cells and increased in tubular and podocytes in diabetic nephropathy.

### Liver

Aging and age-related diseases exhibit markers associated with senescence. Studies on aging liver cells in mice have shown the presence of aging markers, such as TAF54, nuclear hypertrophy, SADS, LINE-1, and SASP factors (107). While understanding of senescence in human liver cells is less advanced than in mice, aging markers such as SA-β-gal have been found in patients with liver cirrhosis (108). Additionally, the expression of CDKN1A and CDKN2A, as well as the presence of TAF, has been observed in non-alcoholic fatty liver disease, and TAF has also been reported in alcoholic liver disease (109). Liver stellate cells in liver injury models also exhibit senescence markers, including SA-β-gal activity and expression of Cdkn1a, Cdkn2a, and SASP components (110).

### Lung

CDKN2A is considered a major marker associated with lung aging. Various mouse models have been developed to study how Cdkn2a-driven lung cell senescence responds to external stressors (111). CDKN1A, p53, and γ-H2AX are also upregulated with aging in both mouse and human lungs (112). Furthermore, research indicates that SERPINE1 induces senescence in mouse alveolar type II (ATII) cells through activation of the p53-CDKN1A-RB1 pathway in fibrotic lung diseases (113). Upregulation of GDF15 and TNFRSF1B in plasma is related to susceptibility to pulmonary interstitial abnormalities. In idiopathic pulmonary fibrosis, another marker of senescent fibroblasts is enhanced expression of BCL2L2, while pro-apoptotic proteins BAK1 (114).

### Pancreas

It has been reported that the proportion of SA-β-gal+ cells in human islets increases with age (115). Type 2 diabetes significantly elevates this proportion, accompanied by higher levels of the DNA damage marker TP53BP1 (116). Aging markers such as Cdkn1a, Cdkn2a, and loss of nuclear Hmgb1 are also found in α-cells of NOD mice, though at a lower rate compared to β-cells within the same mouse (117).

### Skin

Skin aging is influenced by environmental factors such as ultraviolet radiation, making the establishment of reliable aging markers challenging (118). Features of skin aging can be identified through the expression of SA-β-gal, α-fucosidase, CDKN2A, CDKN1A, and lipofuscin in the epidermal and dermal layers (37). These expression patterns are associated with low levels of MKI67 and only a slight shortening of telomeres. Another common feature of skin aging is chromatin distortion, characterized by nuclear foci of TP53BP1 and PML (119). In sun-damaged skin, melanocyte aging is most pronounced, confirmed by increased expression of CDKN2A, a rise in TAF numbers, and loss of nuclear HMGB1 (120).

### Breast

In the mammary stroma of aged mice (25-32 months old), Cdkn2a expression is upregulated approximately 20-fold (121). Immunostaining reveals an increased frequency of ductal cells expressing the SASP factor COX2 with age. Other inflammatory features, such as the crown-like structures of macrophages and increased levels of Cxcl1 and Il6, are also elevated in the mammary glands of aged mice (122).

### Ovary

Research on ovarian aging is primarily conducted in animal models (123). In the ovaries of aged reproductive mice, characteristics of senescent cells include increased expression of Cdkn2a with age (124). Besides natural physiological aging, genotoxic drugs can accelerate reproductive aging, such as the chemotherapeutic agents cisplatin and doxorubicin, which increase SA-β-gal and CDKN2A expression and trigger increases in SASP components such as IL-6, Ccl2, and Tgfb1 (125). In genetic mouse models of accelerated ovarian aging, levels of Cdkn2a, Cdkn1a, Il1a, and Il1b are elevated (126). Additionally, in mouse models of ovarian insufficiency, the formation of SAHF in the ovaries is observed, marked by increased staining for H3K9me3 and CBX5, along with elevated protein expression of HMGA1 and HMGA2 (127).

### Placenta

Developmental senescence is a programmed and transient cellular aging process crucial during embryonic development in mammals (128). During trophoblast differentiation into large multinucleated syncytiotrophoblasts and extravillous trophoblasts, several aging features are exhibited, including loss of replicative potential, formation of SAHF, and TNF mediating immune surveillance and tissue remodeling (129). Compared to normal fetal placentas, restricted fetal placentas have shorter telomeres and higher levels of CDKN1A and EEF1A1 (130).

## Signaling pathways regulating cellular senescence

**Figure 3:**
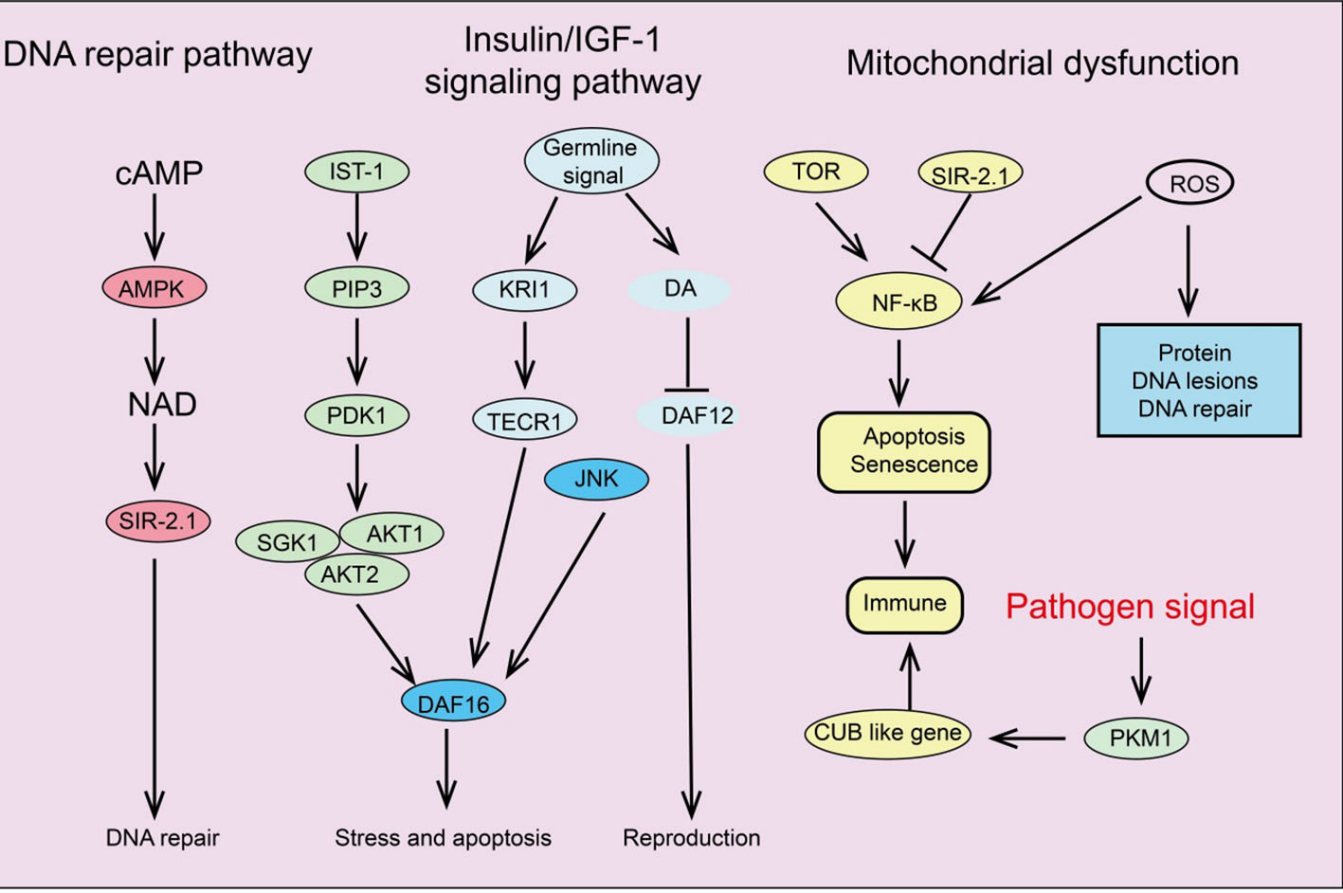
Summary of signaling pathways regulating cellular senescence.

Several key signaling pathways associated with aging have been identified, including the insulin/IGF-1 signaling pathway (IIS), mTOR, AMPK, NF-κB, and Sirtuins pathways. These signaling pathways regulate glucose, amino acids, cAMP, and NAD+ levels by sensing nutrients or metabolic products, forming complex networks related to longevity and aging (**Figure 3**).

### Insulin/IGF-1 signaling pathway

The IIS signaling pathway was the first pathway identified in model organisms as being associated with aging and age-related processes (131). Insulin and insulin-like peptides (ILP) bind to insulin receptors on the surface of target cells, triggering IIS signaling and initiating an intracellular kinase cascade that ultimately activates AKT kinase (132). Activation of AKT leads to the phosphorylation of downstream transcription factor FOXO, inhibiting its transcriptional function and thus promoting cell survival, growth, and proliferation (133). Additionally, the IIS signaling pathway interconnects with other pathways such as mTOR and AMPK, forming a complex network that regulates longevity and aging. Increasing evidence suggests that the GH/IGF-1 signaling pathway plays a crucial role in regulating aging and disease (134). In mammals, growth hormone induces the liver to release IGF-1, which activates insulin receptor substrate molecules (IRS), the PI3K-Akt pathway, and the MAPK signaling cascade by binding to high-affinity IGF-1R on the cell surface, initiating complex intracellular signaling that controls various functions, including mTOR activity and FOXO translocation (135).

### mTOR signaling pathway

mTOR is an evolutionarily conserved serine/threonine kinase that controls processes like protein and lipid synthesis or autophagy (136). Increasing evidence indicates that mTOR signaling influences lifespan and aging. mTORC1 inhibitor rapamycin can reduce mRNA levels of IL-6 and other cytokines and selectively inhibit the translation of membrane-bound cytokine IL-1A (137). The reduction of IL-1A decreases NF-κB transcriptional activity, thus controlling much of the SASP (138). Additionally, in aging mice and cells, two important DNA damage repair proteins, MGMT and NDRG1, are negatively regulated by mTORC1. Notably, the IIS pathway and mTOR are interconnected in regulating aging; for instance, IIS activates mTORC1 via AKT, while mTORC1 can negatively regulate IIS by inhibiting insulin receptor substrate 1 (IRS-1) through S6K (139). Studies have shown that reducing IIS signaling can extend the lifespan of nematodes, fruit flies, and mice.

### AMPK signaling pathway

AMPK plays a fundamental role in cellular and organismal energy metabolism. ATP consumption activates AMPK signaling, shutting down many energy-consuming reactions like protein and lipid synthesis (140). Studies indicate that the ability to activate AMPK signaling declines progressively with age, significantly impairing effective cellular homeostasis and promoting the aging process (141). Therefore, impaired activation of AMPK signaling disrupts downstream signal network functions, leading to intracellular homeostasis maintenance issues. Research shows that AMPK signaling regulates complex networks, including FOXO, mTOR/ULK1, and SIRT1 pathways, by modulating downstream pathways (142). Many studies suggest that IIS signaling inhibits AMPK activation, reducing its downstream activity. Additionally, AMPK can activate ULK1, inhibiting mTOR signaling that enhances autophagy. As aging progresses, the decline in AMPK signaling interferes with the maintenance of autophagy and cellular protein homeostasis (143). Given the crucial role of AMPK in energy metabolism, age-related mitochondrial homeostasis deterioration might also result from inefficient AMPK activation. Moreover, AMPK signaling can inhibit the NF-κB system, controlling inflammation and immune responses, which are disrupted with aging (144). In summary, AMPK activation is associated with many lifespan-extending pathways, such as inhibiting inflammation, suppressing IIS and mTOR signaling, stimulating Sirtuin signaling, and preventing mitochondrial dysfunction. AMPK activators play a vital role in slowing human aging (145).

### NF-κB signaling pathway

The NF-κB system is an ancient host defense system involved in immune responses and reacts to various external and internal danger signals such as oxidative stress, hypoxia, and genotoxic stress (146). Interestingly, some longevity-related genes, such as SIRT1, SIRT6, and the FOXO family, can delay aging and extend lifespan by inhibiting NF-κB signaling (147). Due to numerous interactions between the NF-κB signaling pathway and other signal networks, inhibiting the entire NF-κB system could be detrimental, making the development of specific inhibitors targeting different branches of the NF-κB signaling cascade essential (148).

### Sirtuins pathway

Sirtuins are crucial in regulating various cellular processes, including metabolism, mitochondrial homeostasis, oxidative/antioxidant balance, and aging (149). SIRT1 can catalyze the deacetylation of histones H1, H3, and H4, as well as non-histone proteins such as p53, Ku70, the FOXO family, PGC1α, PPAR-γ, and NF-κB, thus extensively participating in regulating cellular senescence and organismal lifespan (150, 151). Additionally, SIRT1 integrates multiple signaling pathways involved in aging regulation. SIRT1 negatively regulates NF-κB signaling, which is activated in aging-related diseases, promoting the aging process (152). Studies have shown that SRT1720 and resveratrol inhibit NF-κB activity by activating SIRT1, playing essential roles in anti-inflammatory and anti-aging processes (153). Furthermore, SIRT1 and AMPK closely interact in regulating energy metabolism and aging, mutually enhancing each other’s activity. For example, AMPK delays cellular senescence by inducing NAD+/SIRT1 and upregulating autophagy. SIRT1 also interacts with mTOR in aging regulation by modulating autophagy function, such as restoring autophagy damage induced by oxidative stress by blocking the mTOR pathway, and improving the survival rate of embryonic stem cells (154). Upregulating SIRT1 can influence these pathways’ regulation of aging and age-related diseases (155). Therefore, using small molecule agonists to activate SIRT1 may be an effective strategy for extending lifespan and improving age-related diseases.

## Functions of cellular senescence

### Embryogenesis and development

Transcription factors INK4b and p66Shc have been found in the embryonic brains of chickens and cows, suggesting that senescence plays a role in regulating embryonic development (156). In mammalian embryos, senescence can occur in various locations such as the limbs, nervous system, and endoderm of the intestine (157). In developing kidneys, aggregated senescent cells promote mesonephros degeneration through macrophage-mediated phagocytosis (158). Senescence markers such as p21, p27, and p15 can be detected in embryonic mouse kidneys.

### Wound healing

Wound healing is a physiological response to tissue injury, involving inflammation, tissue formation, and remodeling, where cellular senescence plays a crucial role (159). During skin wound healing, matrix cell protein CCN1 induces fibroblast or myofibroblast senescence by activating DDR and ROS-p16 signaling, thereby reducing fibrosis (160). However, cellular senescence can also interfere with wound healing. Extracellular vesicles from senescent mesenchymal stem cells (MSCs) inhibit wound healing by downregulating miR-146a (161). These findings suggest that while transient cellular senescence can promote tissue repair, the prolonged presence of senescent cells can impair tissue healing.

### Cancer progression

Cellular senescence may have a dual role in cancer. Oncogene activation, tumor suppressor gene loss, and irreparable DNA damage induce not only apoptosis but also senescence to prevent tumorigenesis. Temporary damage can lead to cell cycle arrest, thereby preventing oncogenic mutations from being passed to daughter cells and accelerating their immune clearance (162). DDR signaling is a primary mechanism of oncogene-induced senescence. The transcription factor HBP1 has been identified as a novel agonist of p21, functioning by either attenuating p53 degradation or regulating the Wnt-β-catenin-EZH2 signaling pathway independently of p53 (46). TIMP1 is a crucial molecule in determining the senescence effect in prostate cancer; inactivation of TIMP1 promotes tumor metastasis by activating matrix metalloproteinases, leading to the upregulation of pro-cancer secretory factors (39).

### Immunosenescence

In healthy individuals, immunosenescence leads to increased susceptibility to infections, autoimmune diseases, cancer, and chronic inflammation (163). A significant mechanism of immunosenescence is the reduction in autophagy. ROS and DNA damage. Patients with KD exhibit immunosenescence similar to that of elderly individuals, with the resulting chronic inflammation associated with declining renal function, cardiovascular diseases, and infections (164). However, in the absence of immune cell activation, immunosenescence can be beneficial. For example, the senescence of CD4+ T cells can facilitate better acceptance of donor organs in kidney transplant recipients. Naive B cells are significantly reduced in elderly individuals, impairing their response to pathogens (165). They release pro-inflammatory factors that promote chronic inflammation and carry excess ROS, reducing their proliferative capacity (166). Interestingly, mature macrophages share inherent phenotypic similarities with senescent cells in terms of signaling pathways, gene expression, metabolism, and organelle function (167). Thus, the senescence-like phenotype of macrophages may represent a physiological activation state in response to external challenges rather than true senescence. The interaction between true senescent cells and senescence-like macrophages warrants further investigation.

### Stem cell senescence

Stem cells play a vital role in tissue and organ regeneration by releasing paracrine factors to repair damage (168). However, risk factors, such as aging, can lead to SCS and dysfunction, thereby triggering age-related diseases. The underlying mechanisms of SCS are complex and diverse, including oxidative stress (ROS), non-coding RNAs, miRNAs, and others. Transcription factors such as NANOG, GATA6, and SOX11 also regulate SCS from different sources (169). Some studies suggest that autophagy inhibits the senescence of mesenchymal stem cells (MSCs), while high glucose-induced autophagy exacerbates bone marrow SCS (170).

### Redox regulation of senescence

Excessive ROS or antioxidant deficiencies leading to oxidative stress can induce cellular senescence. Many factors, such as high glucose, radiation, and oncogene expression, disrupt redox balance, causing DNA damage (171). For instance, melanocytes and keratinocytes with autophagy defects undergo senescence when exposed to excess ROS; lactate and fumarate induce senescence in fibroblasts and renal carcinoma cells through oxidative stress. Besides directly triggering the DNA damage response (DDR), ROS also influences Akt signaling, a crucial pathway in cellular senescence, by regulating the expression of various miRNAs (172). Redox balance is essential for maintaining telomere integrity, with telomere shortening and loss hindering DNA replication and leading to replicative senescence (173). Compared to healthy fibroblasts, senescent cells exhibit higher ROS levels, slower proliferation, impaired bioenergetic function, increased DNA damage, and DDR (174).

## Anti-senescence drugs and methods

### Metformin

**Figure 4:**
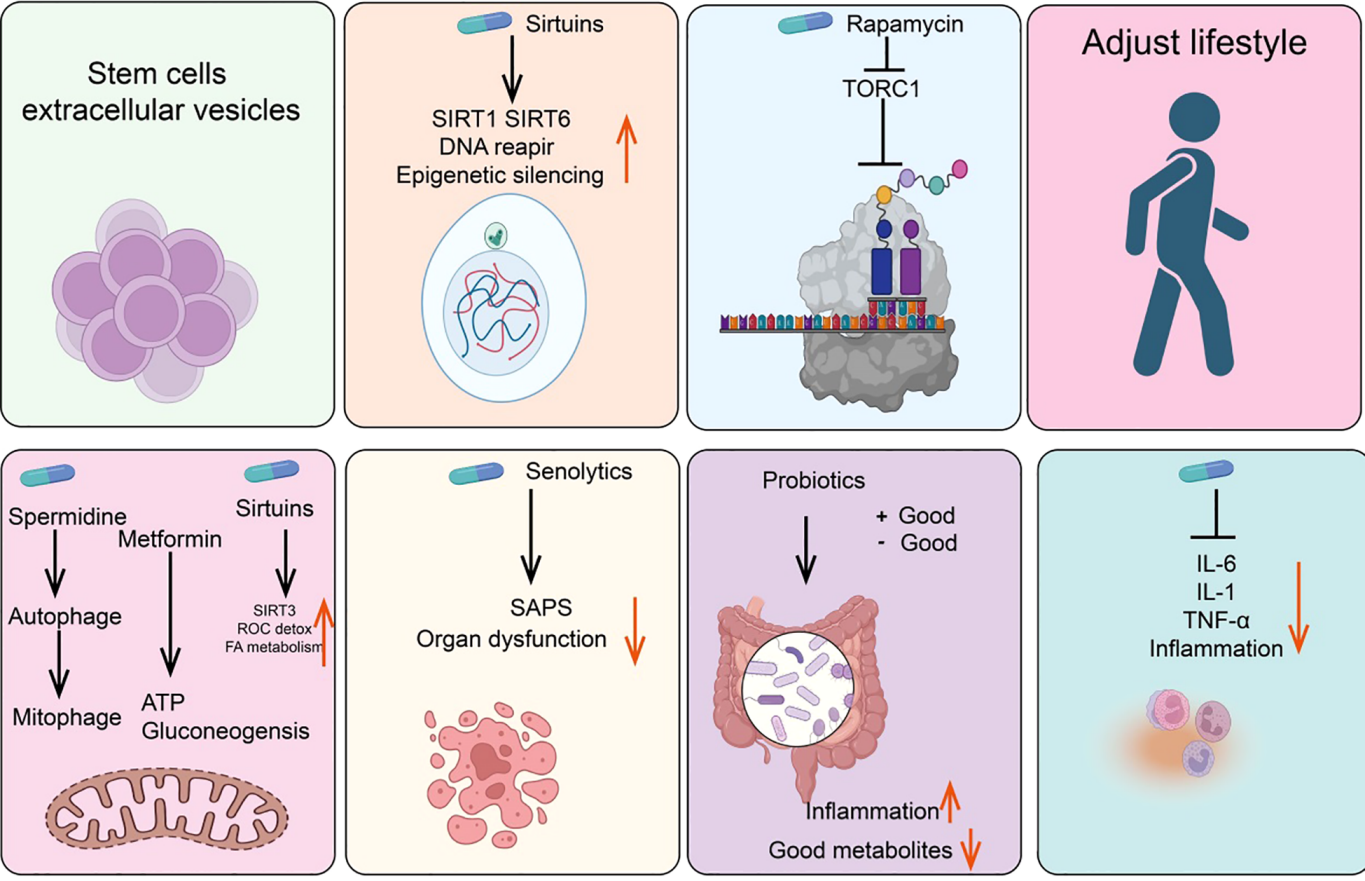
Summary of anti-senescence drugs and strategies.

Metformin is the preferred oral hypoglycemic agent for treating Type 2 diabetes. Although its exact mechanism for lowering blood glucose is not fully understood, one possible pathway involves inhibiting mitochondrial respiratory complex I, thereby reducing ATP synthesis and activating AMP-activated protein kinase (AMPK) (175, 176). Additionally, research indicates that the dimeric form of metformin, subform in, has significant copper-chelating activity and exhibits strong anti-inflammatory effects (177, 178). Other potential mechanisms include partial inhibition of the respiratory chain, which reduces the production of ROS, thereby decreasing the release of tissue-damaging factors and consequently reducing inflammation and accumulation of senescent cells (179, 180). Studies have shown that oral metformin (as opposed to intraperitoneal injection) can modify the gut microbiome of high-fat diet mice and reduce tumor cell growth (181) (**Figure 4**).

### NAD+/sirtuins

Research in the 1990s revealed that yeast Sir2 and its homologs (sirtuins) in higher organisms can delay aging and extend lifespan in yeast, nematodes, fruit flies, and mice (182, 183). Notably, recent studies have found that rare SIRT6 variants are frequently present in centenarians, either increasing gene expression or altering protein amino acids (184, 185). Furthermore, sirtuins are involved in NAD+-dependent histone deacetylase activity and epigenetic regulation (186). NAD+ levels decline with age. In addition to NAD+ supplements, resveratrol, quercetin, and natural flavonoid compounds such as piceatannol can also directly activate the mammalian deacetylase SIRT1, extending the lifespan of nematodes (187).

### Glucagon-like peptide-1

GLP-1 is an incretin hormone produced by the gut after food intake, playing a key role in glucose homeostasis by stimulating insulin secretion and inhibiting glucagon secretion (188, 189). Glucagon and GLP-1 are related but distinct peptides derived from different regions of the proglucagon molecule. Glucagon is produced by the pancreas during fasting, whereas GLP-1 is produced by the gut in response to feeding (190, 191). GLP-1 affects the gut, pancreas, heart, brain, and liver, and GLP-1 receptors have been identified in these organs (192). Current GLP-1-based drugs include GLP-1 receptor agonists such as semaglutide, dulaglutide, abiglutide, exenatide, liraglutide, lixisenatide, and terzepatide, which have received FDA approval and are widely studied (193, 194). Importantly, GLP-1 receptor agonists can slow or even reverse brain aging-related changes in mice.

### Rapamycin/TORC1

Rapamycin, originally isolated from the soil bacterium discovered on Easter Island, is now widely used as an immunosuppressant (195). Rapamycin functions by inhibiting the activity of the TORC1 and later found in mammals as a key regulator of nutrient sensing and cell growth (196, 197). TORC1 activity is inhibited by cell signaling under nutrient deprivation, preventing cell growth and activating autophagy (198, 199). Studies show that rapamycin not only extends lifespan in young mice but also prolongs lifespan when administered to older mice (200, 201).

### Spermidine/autophagy

Spermidine, a natural polyamine, is the only polyamine known to extend lifespan in nematodes, fruit flies, and mice (202). Additionally, spermidine can prevent metabolic syndrome and obesity induced by a high-fat diet in mice and delay age-related cognitive decline (203, 204). One of the most extensively studied effects of spermidine is its ability to induce autophagy, with its protective effects closely related to this capability (205). Aging is associated with a decline in autophagic function, and experimental inhibition of autophagy is sufficient to accelerate aging in mice. Indeed, autophagy is considered one of the key mechanisms through which caloric restriction or TOR inhibition prolongs lifespan (206, 207). Interestingly, other longevity-extending pathways or compounds, such as NAD+/sirtuins and spermidine, also act as inducers of both autophagy and mitophagy. Genetic or pharmacological blockade of endogenous spermidine synthesis reduces fasting-induced autophagy in yeast, nematodes, and human cells. Mechanistically, spermidine mediates these effects through autophagy induction and the oligoglycosylation of the translation regulatory factor eIF5A. In summary, the polyamine-hydroxybutyrylation axis has become a phylogenetically conserved metabolic control center for fasting-induced autophagy enhancement and lifespan extension (204).

### Senolytics

Senolytics are compounds that can selectively eliminate senescent cells without damaging normal cells (208). Subsequent studies have shown that senescent cells also accumulate in aging organisms and that their numbers can be reduced with caloric restriction (209). Since 2004, there has been an effort to identify drugs capable of selectively clearing senescent cells—known as senolytics (210, 211). Current senolytics include natural flavonoid quercetin, dasatinib, flavonoid fisetin, and natural grape seed extract proanthocyanidin C1 (212, 213). Additionally, bisphosphonates and navitoclax, have also been found to effectively clear senescent cells. Drugs that selectively induce the clearance of senescent cells (senolytics) and those that attenuate the tissue-destructive secretory phenotype of certain senescent cells (senomorphics) appear to delay or alleviate the onset of various diseases, including but not limited to endocrine disorders such as diabetes, complications of obesity, age-related osteoporosis, and cancer, as well as atherosclerosis, chronic kidney disease, and neurodegenerative diseases (214).

## Probiotics/intestinal microbiome

The human gut harbors a diverse microbiota that can exert either positive or negative effects on health (215). Probiotics are beneficial bacteria within the gut that promote health. Variations among microbial species may influence immune responses through different bacterial metabolites entering the bloodstream or by affecting the gut barrier in various ways (216). Studies involving fecal transplants from elderly donor mice to young recipient mice have demonstrated that donor inflammatory and health conditions can be transmitted to the recipients (217). Similarly, transplantation of gut microbiota from healthy young mice to prematurely aged mice can extend their lifespan (218).

## Anti-inflammatory drugs

Anti-inflammatory drugs, including corticosteroids, aspirin, and ibuprofen, are commonly used analgesics (219). Systemic chronic inflammation that increases with age is a significant driver of the aging phenotype, making anti-inflammatory drugs potential agents for mitigating age-related diseases (220). In this context, supplementation with the NAD+ precursor nicotinamide riboside (NR) or using antibodies to block tumor necrosis factor alpha (TNF-α) receptors can alleviate aging phenotypes (221). An interesting question is whether reducing inflammation affects DNA methylation clocks, which is relevant for assessing the rate of biological aging (222).

## Stem cells and extracellular vesicles

Stem cells and their extracellular vesicles can also effectively clear senescent cells (223). For instance, MSCs can alleviate aging in elderly mice and improve cardiac function, pluripotent stem cells can prevent stress-induced cardiomyocyte aging, and human umbilical cord-derived MSCs can protect rat kidneys from acute AKI-induced aging (224). The extracellular vesicles of stem cells have significant anti-aging activity and are less likely to induce rejection or tumor formation compared to parental stem cells (225).

## Lifestyle interventions

Certain lifestyle factors may accelerate aging. For example, sleep deprivation activates DNA damage response (DDR) mechanisms and promotes SASP production, whereas a healthy lifestyle, such as regular exercise and caloric restriction, can delay aging (226). Studies in animals and humans have shown that regular moderate-intensity exercise in elderly individuals has a positive impact on immune aging and age-related diseases by modulating mitochondrial function, inflammation, Long-term exposure to external stressors, such as temperature fluctuations, and nutrient deprivation, may activate adaptive homeostatic mechanisms via the NRF2-KEAP1 signaling pathway (227). Adjusting these stress exposure levels could become a novel strategy to improve lifespan and potentially exert beneficial effects on cellular aging (228).

## Role of m6A modification in cellular senescence

**Figure 5:**
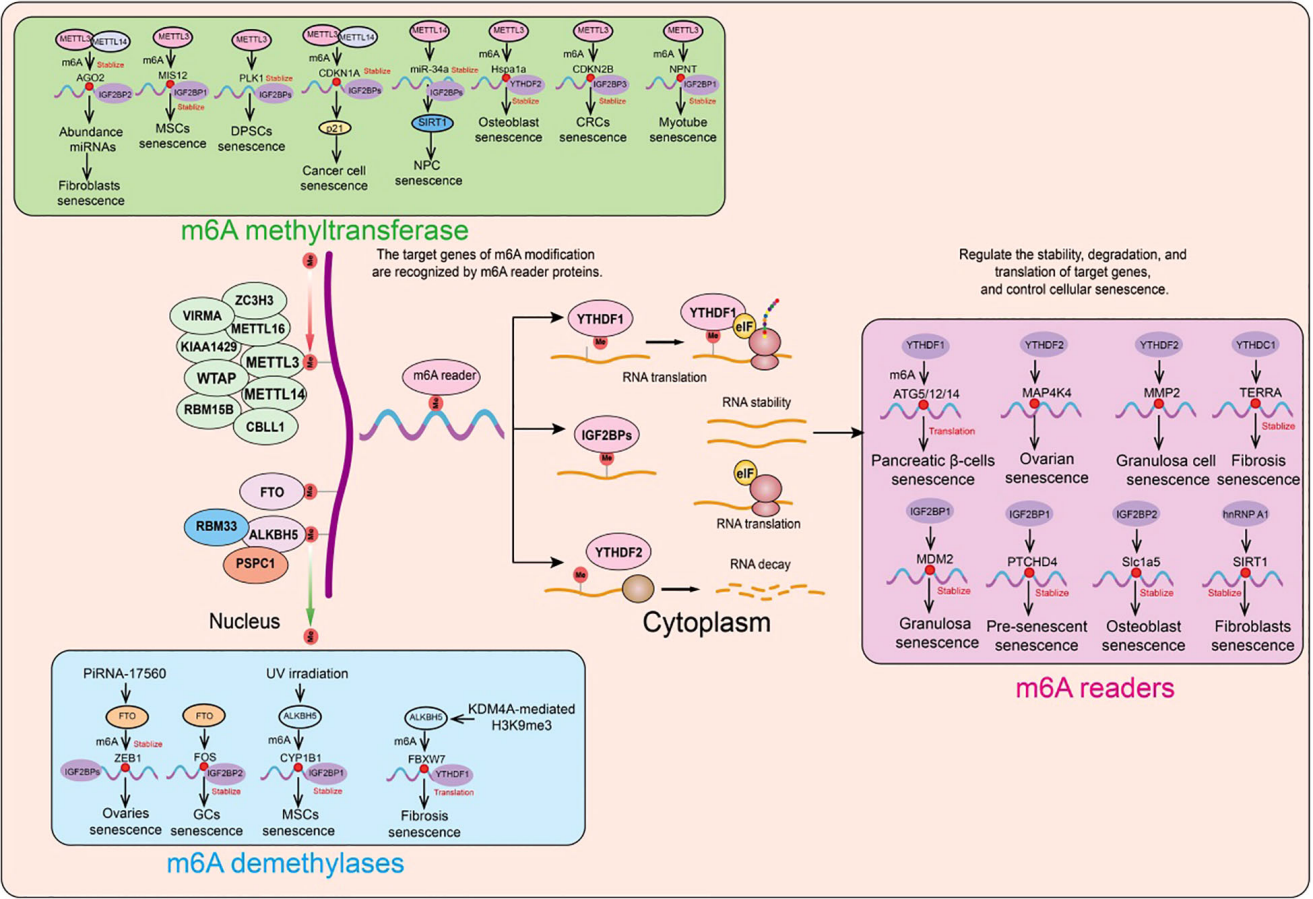
Functions and mechanisms of m6A regulatory factors in different cellular senescence contexts. m6A regulatory factors regulate the expression of target genes through different mechanisms, thereby influencing a series of aging-related events.

We have systematically summarized the roles and mechanisms of m6A modification regulatory factors in cellular senescence (**Figure 5**).

### Role of m6A methyltransferases in cellular senescence

A prospective study collected liver, heart, and skeletal muscle samples from 8 young and 8 elderly macaques. Phenotypic analysis showed that compared to the young macaques, the elderly macaques had significantly increased lipid accumulation in their liver, heart, and skeletal muscle, increased expression of SASP (senescence-associated secretory phenotype) related genes, and decreased levels of lamin B1 protein (a marker of cellular senescence). Furthermore, the researchers performed m6A-seq and RNA-seq on the liver, heart, and skeletal muscle tissues from both young and elderly macaques to describe changes in m6A modification during aging. Consistent with previous studies, m6A consensus sequences (such as the highly enriched GGACU) were present in the liver, heart, and skeletal muscle tissues, with a significant enrichment near the stop codon. The overall distribution pattern of m6A modifications was similar in the liver, heart, and skeletal muscle. In both young and elderly liver, heart, and skeletal muscle samples, the researchers found that the m6A modifications and transcriptional features of the heart and skeletal muscle were more similar to each other, a phenomenon that is related to the similar tissue morphology and function of the heart and skeletal muscle (229).

Recent studies have revealed that m6A modification and methyltransferase-like 3 (METTL3) expression are reduced in elderly osteoporotic patients. Further *in vitro* experiments showed that METTL3 inhibits osteoblast senescence, and osteoblast dysfunction affects bone metabolic balance. Using MeRIP-seq and mRNA-seq, it was found that METTL3 regulates the stability of the Hspa1a transcript. During the inhibition of osteoblast senescence by METTL3, YTH N6-methyladenosine RNA binding protein 2 (YTHDF2) delayed the decay of Hspa1a mRNA (230).

In human mesenchymal stem cells (MSCs), the absence of METTL3 leads to decreased m6A modification and MIS12 expression, thereby accelerating senescence (231). The m6A-binding protein (reader) IGF2BP2 binds and stabilizes m6A-modified MIS12 mRNA, countering premature senescence in human MSCs (231). The nuclear lamina protein Lamin A interacts with METTL3 and METTL14, stabilizing these two proteins. Knockdown of METTL3 or METTL14 accelerates senescence, whereas overexpression of METTL14 can attenuate replicative senescence in normal human fibroblasts and improve the senescence phenotype of fibroblasts in premature aging mouse models. Additionally, overexpression of METTL14 in senescent human fibroblasts alleviates abnormal nuclear envelope defects and restores the expression of the heterochromatin marker H3K9me3 (232).

To investigate the specific role of m6A modification in shaping the transcriptomic features associated with senescence, researchers first analyzed m6A-modified RNA in senescent and proliferating cells. They discovered that the METTL3/METTL14 complex integrates m6A modifications into PTCHD4 mRNA; the addition of m6A stabilizes PTCHD4 mRNA and increases the production of PTCHD4. Using MeRIP RT-qPCR and eCLIP analyses, this m6A modification was localized to the last exon of PTCHD4 mRNA. Further studies revealed that IGF2BP1, rather than other m6A readers, is responsible for the stabilization and increased abundance of m6A-modified PTCHD4 mRNA. Silencing the transmembrane protein PTCHD4 enhanced growth arrest and DNA damage in pre-senescent cells, making them more susceptible to senescence and apoptosis. These findings suggest that the m6A modification of PTCHD4 mRNA promotes the production of PTCHD4, a protein associated with the survival of senescent cells (233).

In human dental pulp stem cells (DPSCs), METTL3/METTL14 have been shown to catalyze m6A modification in the 3’UTR of CDKN1A mRNA, which encodes the cell cycle-dependent kinase inhibitor p21, thereby promoting cell cycle arrest in oxidative stress-induced senescence of human cancer cells (234, 235). Cellular senescence is typically characterized by cell cycle arrest and is a major marker and driver of tissue degeneration and aging. Emerging evidence suggests that m6A RNA regulation is involved in the senescence process, potentially through altering the levels of cell cycle regulators. Notably, knockdown of METTL3 or METTL14 reduces p21 protein levels without affecting its overall mRNA levels, indicating that m6A plays a role in enhancing p21 translation. The tumor suppressor p53, an upstream factor of p21, is involved in m6A-related senescence regulation (235). Similarly, other cell cycle-related factors are regulated by m6A in human stem cell aging. For example, the knockdown of METTL3 accelerates senescence in human DPSCs, evidenced by increased SA-β-gal staining and disrupted cell cycle progression. This process may be mediated by the upregulation of PLK1, a key cell cycle regulator, at both mRNA and protein levels due to reduced m6A modification (236). Additionally, it has been confirmed that METTL3/METTL14-mediated m6A modification promotes AGO2 mRNA stability and subsequent expression of mature miRNAs. Knockdown of METTL3 or METTL14 also accelerates fibroblast senescence. Although this study integrates m6A regulation with miRNA processing related to aging, future mechanistic research will be crucial for understanding which m6A readers stabilize methylated AGO2 mRNA and which miRNAs play a predominant role in anti-aging. It has also been reported that m6A-related miRNA processing issues occur in human nucleus pulposus cell (NPC) senescence, as knockdown of METTL14 alleviates TNF-α-induced cell cycle arrest and NPC senescence by inhibiting m6A and miR-34a expression. Mechanistic insights suggest that the interaction between METTL14 and DGCR8 promotes miR-34a processing, with miR-34a targeting SIRT1 to accelerate NPC senescence (237). These findings indicate that m6A-mediated miRNAs and their target mRNAs are involved in cellular senescence. However, this field is still developing and requires further research to fully understand its potential mechanisms and functional consequences.

For instance, in human MSCs, loss of FTO disrupts its interaction with MIS12, leading to reduced MIS12 protein levels (not its mRNA form) and accelerated senescence (238). Further evidence indicates that FTO’s interaction with MIS12 protects MIS12 from proteasomal degradation, though the detailed mechanism remains to be explored (239). These findings highlight the significant role of m6A methyltransferases and demethylases (such as METTL3, METTL14, and FTO) in regulating cellular senescence. Age-related osteoporosis is primarily caused by impaired osteoblast function, leading to reduced bone mass and disrupted bone remodeling processes. A recent study revealed the role of METTL3 in osteoblast senescence. The study found reduced expression of METTL3 in osteoblasts. Further *in vitro* experiments showed that METTL3 can inhibit osteoblast senescence, while osteoblast dysfunction affects bone metabolic balance. Mechanistic studies indicate that METTL3 exerts its effects by regulating the stability of Hspa1a transcripts (230). During METTL3-mediated inhibition of osteoblast senescence, YTHDF2 is involved in the stabilization of Hspa1a mRNA through m6A modification. Another study showed that in colorectal cancer (CRC) cell replicative senescence, the m6A modification level is significantly increased, associated with upregulation of the methyltransferase METTL3. Functional experiments demonstrated that METTL3 regulates CRC cell senescence in an m6A-dependent manner. Further m6A-seq data analysis identified a key senescence-regulating factor CDKN2B, and found that METTL3 can regulate CDKN2B expression. Mechanistic studies revealed that METTL3-induced m6A methylation at A413 in the CDKN2B CDS recruits IGF2BP3, affecting its binding with CCR4, thereby stabilizing CDKN2B mRNA (240). Additionally, upregulation of m6A-modified E2F1 expression binds to the -208 to -198 region of the CDKN2B promoter, promoting CDKN2B transcription. Researchers used the METTL3 inhibitor STM2457 to target m6A modification in cells, thereby inhibiting CRC cell senescence (240).

A recent significant study revealed the role and molecular mechanisms of m6A methylation in tissue aging. By analyzing liver, skeletal muscle, and heart tissues from young and old crab-eating monkeys, researchers systematically mapped m6A epi transcriptomics and corresponding transcriptomics, revealing the relationship between m6A modification and gene expression homeostasis, and the aging regulatory patterns in different tissues (241). Compared to the liver and heart, the overall m6A modification level is reduced in skeletal muscle, with decreased expression of the core methyltransferase METTL3. Using CRISPR/Cas9 technology, researchers constructed METTL3 knockout myotube cells derived from human embryonic stem cells, finding that METTL3 deficiency leads to atrophy, apoptosis, and accelerated aging of myotubes, consistent with the phenotype of aging skeletal muscle. Mechanistic studies identified NPNT as a downstream effector of METTL3, maintaining skeletal muscle cell homeostasis (241). Lentiviral-mediated METTL3 or NPNT re-expression can partially delay aging in human myotube cells. Finally, experiments with METTL3 enzyme activity inhibitors and overexpression of METTL3 enzyme activity mutants confirmed that METTL3 promotes NPNT expression through m6A catalytic activity-dependent mechanisms, maintaining myotube cell homeostasis. Additionally, the m6A-binding protein IGF2BP1 was found to bind and stabilize m6A-modified NPNT mRNA (241).

### The role of m6A demethylases in cellular senescence

In human ovarian granulosa cells, downregulation of FTO increases global m6A levels and enhances the stability of FOS mRNA, thereby promoting the aging of granulosa cells. In human nucleus pulposus cells (NPCs), METTL14 interacts with DGCR8 and facilitates the generation of miR-34a-5p through an m6A-dependent mechanism, accelerating NPC aging by targeting and inhibiting SIRT1 (242). During RAS-induced fibroblast cell aging, the genomic regions bound by METTL3 and METTL14 are redistributed, leading to the formation of chromatin loops between the promoters and enhancers of senescence-associated secretory phenotype (SASP) genes, thereby promoting SASP transcriptional activation through an m6A-independent mechanism (243). Research has shown that extracellular vesicle-derived piRNA-17560 from aging neutrophils enhances the expression of FTO in breast cancer cells (244). Through decreasing m6A RNA methylation, further stabilizes and increases the expression of ZEB1 transcripts, leading to chemoresistance and EMT in tumor cells (244). A 2021 study reported significant downregulation of FTO in human FF and GCs from elderly donors, as well as similar m6A modification and FTO expression dysregulation in aging human ovaries. In human ovarian granulosa cell lines, FTO knockdown accelerates GC aging, while FOS inhibition alleviates this aging, confirming that FOS mRNA is a key downstream effector of m6A (245). Further analysis indicated that IGF2BP2 is responsible for stabilizing m6A-marked FOS mRNA (245).

Previous studies have linked mesenchymal stem cell (MSC) aging to the development of senescence-related diseases such as osteoarthritis (OA). A recent study found that in aging MSCs induced by H2O2 stimulation or UV irradiation, m6A levels are significantly increased, while ALKBH5 expression is reduced. Downregulation of ALKBH5 increases the m6A modification levels of CYP1B1, recruits IGF2BP1 to maintain CYP1B1 mRNA stability, and induces mitochondrial dysfunction, promoting MSC aging (246). Additionally, ALKBH5 knockout in MSCs exacerbates spontaneous OA in mice, whereas ALKBH5 overexpression improves MSC efficacy in OA. ALKBH5 expression is significantly elevated in intervertebral disc degeneration and NPC aging, primarily due to reduced KDM4A-mediated H3K9me3 modification. ALKBH5 promotes NPC aging by demethylating DNMT3B transcripts and inhibiting YTHDF2-mediated degradation (247). Moreover, 1-NP exposure induces lung fibrosis in mice by damaging telomeres and inducing cellular aging. 1-NP exposure upregulates m6A modification of FBXW7 mRNA, a process dependent on ALKBH5-YTHDF1, thereby increasing FBXW7 translation efficiency and protein expression (248). Downregulation of ALKBH5 results in increased m6A modification of FBXW7, with YTHDF1 promoting FBXW7 mRNA translation. Studies suggest that 1-NP exposure, by increasing FBXW7 levels, enhances TRF2 ubiquitination and proteasomal degradation, mitigating 1-NP-induced TRF2 degradation and cellular aging (248).

### The role of m6A reader proteins in cellular senescence

As a well-known m6A recognition protein, YTHDF1 is upregulated by HIF-1α transcriptional regulation, promoting the expression of autophagy-related proteins (e.g., ATG5, ATG2A, and ATG14), thus facilitating hypoxia-induced apoptosis and aging of pancreatic β-cells (249). A recent study in an intestinal stem cell model found that BuGZ and its phase separation properties regulate intestinal stem cell proliferation during aging and injury, thereby affecting intestinal homeostasis and the lifespan of Drosophila. Mechanistic investigations revealed that the m6A reader protein YT521-B, under the regulation of BuGZ and its phase transition characteristics, conducts transcriptional regulation and mediates intestinal stem cell proliferation, intestinal homeostasis, and lifespan through the downstream MAPK signaling pathway (250). In ovarian aging, Ras reduces the expression of RNA-m6A reader YTHDF2 by stimulating reactive oxygen species (ROS) production. Knockdown of YTHDF2 significantly enhances the mRNA stability of MAP2K4 and MAP4K4, promoting their expression and accelerating ovarian aging through activation of the NF-κB signaling pathway and upregulation of SASP expression (251). In granulosa cells, increased m6A modification of total RNA and reduced expression of the demethylase FTO and its target gene MMP2 have been observed. Knockdown of FTO and MMP2 promotes granulosa cell aging, while their overexpression delays aging. YTHDF2 and FTO can bind to the mRNA of MMP2, regulating the downstream extracellular signal-regulated kinase (ERK) pathway and thus promoting granulosa cell aging (252). Recent research has highlighted the significant role of YTHDC1 in cellular aging and lung fibrosis. YTHDC1 is expressed in alveolar type II cells (AECII) and its expression decreases significantly during fibrosis. Exogenous overexpression of YTHDC1 can alleviate aging and fibrosis in lung cells, whereas YTHDC1 knockout exacerbates disease progression in mice. Mechanistic studies reveal that YTHDC1 promotes the interaction between TopBP1 and MRE11 through its N-terminal domain, activating ATR and facilitating DNA damage repair. This function of YTHDC1 is crucial for maintaining genomic stability and protecting the lungs from stress-induced aging and fibrosis (253). TERRA forms R-loops by invading telomeric DNA. Both excessive and insufficient R-loop formation can lead to telomere instability, requiring precise regulation of TERRA levels. YTHDC1 has been reported to directly bind to TERRA transcripts, enhancing TERRA stability and thereby maintaining telomere stability (253).

In the ovary, 3-nitropropionic acid (3-NP) treatment significantly reduces IGF2BP1 expression. IGF2BP1 enhances MDM2 mRNA stability in an m6A-dependent manner, regulating the vitality, proliferation, cell cycle, and aging of granulosa cells (GCs) (254). Overexpression of IGF2BP1 can partially rescue 3-NP-induced GC damage, while ectopic expression of MDM2 can alleviate GC dysfunction caused by 3-NP or IGF2BP1 knockdown. Additionally, the METTL3/METTL14 complex introduces m6A modifications into PTCHD4 mRNA, stabilizing it and increasing PTCHD4 production (233). IGF2BP1 further maintains the stability and abundance of PTCHD4 mRNA, enhancing growth arrest and DNA damage in pre-senescent cells, making them more sensitive to aging and apoptosis (233). In osteoporosis patients, the expression of the m6A reader protein Igf2bp2 is significantly upregulated. Increased Igf2bp2 expression promotes osteoblast aging by enhancing Slc1a5 mRNA stability and inhibiting cell cycle progression. Furthermore, Mettl3 has been identified as a Slc1a5 m6A methylation protein, with increased m6A modification levels leading to osteoblast aging, whereas Mettl3 overexpression promotes osteoblast aging (255). Notably, delivery of Igf2bp2 small interfering RNA also induces bone mass increase and reduces bone marrow fat accumulation. hnRNP A1 directly interacts with the 3’ untranslated region of SIRT1 mRNA, promoting its stability and increasing SIRT1 expression, thereby delaying replicative cellular aging (256). CGP 57380 significantly reduces hnRNP A1 phosphorylation levels in both young and aged fibroblasts, preventing the increase in senescent cells (256).

## The application and challenges of different m6A sequencing methods in cellular senescence

The dynamic regulation of m6A RNA modification plays a crucial role in various biological processes, including cellular senescence and organismal aging. Recent advancements in m6A sequencing technologies have greatly improved our understanding of how m6A modifications affect gene expression and cellular functions in these contexts. Several sequencing methods, such as MeRIP-seq (257), m6A-seq (245), GLORI (258), miCLIP (259), m6A-SAC (260), m6A-LAIC-seq, m6A-seq2 (261) and photo-crosslinking–based techniques, have been developed to map m6A modifications across the transcriptome with high precision. These methods allow for the identification of m6A-modified RNA molecules and provide insights into the dynamic changes in m6A levels during cellular aging and organismal senescence.

The application of these m6A sequencing technologies in aging research has revealed important insights into the regulation of gene expression during aging. For instance, studies using m6A sequencing have identified key genes involved in cellular stress responses, metabolism, and cell cycle regulation that are regulated by m6A modifications during aging. In addition, m6A modifications have been implicated in age-related diseases, such as neurodegenerative disorders and cancer, highlighting the potential of targeting m6A pathways for therapeutic interventions. However, the application of m6A sequencing methods to aging research also presents several challenges. One major limitation is the difficulty in distinguishing between direct and indirect effects of m6A modifications on gene expression, as m6A modifications can influence RNA stability, splicing, and translation in complex ways. Furthermore, the heterogeneity of aging processes across different tissues and organisms makes it challenging to obtain a comprehensive understanding of the role of m6A modifications in aging.

To overcome these challenges, further improvements in m6A sequencing technologies are needed. New methods that combine m6A sequencing with single-cell RNA sequencing and transcriptome-wide profiling of RNA modifications will allow for more precise analysis of m6A dynamics in individual cells and tissues. Additionally, the development of advanced bioinformatics tools to analyze large-scale m6A sequencing data will be crucial in interpreting the complex regulatory networks involved in aging. Despite these challenges, the application of m6A sequencing in aging research holds great promise for uncovering novel mechanisms of aging and identifying potential targets for anti-aging therapies.

## The intersection between cellular senescence and tumor progression

The emergence of malignant cells is often due to the inability of age-related mechanisms to maintain cellular identity at the genomic and epigenomic levels, which, together with age-associated ecological imbalances, immune surveillance failures, inflammation, and metabolic deviations, promotes tumorigenesis (262). Simultaneously, cancer cells must overcome various age-related mechanisms that typically restrict cellular adaptability, proliferation, and plasticity, such as autophagy suppression, cellular senescence, stem cell depletion, and telomere attrition (263). Senescence markers not only facilitate tumor development but may also, in some cases, prevent it. Age-related metabolic dysregulation may lay the groundwork for metabolic reprogramming in tumor cells; however, cancer cells often activate a distinct set of metabolic alterations driven by oncogenes and genetic disturbances, which impair tumor suppression. Research on biological aging clocks in patients is relatively limited.

## Pro-tumorigenic effects of cellular senescence

Cellular senescence is a state of permanent cell cycle arrest that, although essentially a protective mechanism to prevent cancer, can, in certain contexts, promote malignant tumor progression (264). Senescent cells secrete a large array of pro-inflammatory cytokines, chemokines, proteases, and growth factors (265). Pro-inflammatory factors secreted by senescent cells, such as IL-6, IL-8, and TNF-α, can attract and activate immune cells, leading to chronic inflammation. This chronic inflammatory environment supports tumor cell survival and progression. SASP components, including proteases like MMPs, promote diverse cancer cell invasion and metastasis (266). Additionally, SASP can activate surrounding stromal cells, altering their function and further promoting tumor progression (267). Certain factors secreted by senescent cells can suppress immune system anti-tumor functions, for instance, by inhibiting the activity of cytotoxic T cells and NK cells, allowing tumor cells to evade immune surveillance (268). Furthermore, some SASP factors can enhance the stem cell-like properties of tumor cells, endowing them with greater self-renewal and differentiation capabilities, which increases tumor recurrence and resistance (269). Although the initial intent of cellular senescence is to prevent abnormal cell proliferation and cancer, the accompanying SASP can, in some cases, exacerbate malignant tumor progression. Therefore, effective strategies to clear senescent cells or inhibit SASP are crucial areas of current cancer research.

## Anti-tumor effects of cellular senescence

Cellular senescence is a mechanism characterized by permanent cell cycle arrest, which plays a crucial role in suppressing malignant tumor progression. In certain contexts, cellular senescence prevents tumor initiation and development through several mechanisms that inhibit cell proliferation (270). Senescent cells cease to divide and exit the cell cycle, thereby halting the proliferation and dissemination of potentially oncogenic cells (271). This mechanism effectively confines harmful cells to a quiescent state, preventing their evolution into malignant tumors. Facilitating immune clearance: senescent cells secrete specific factors that attract NK cells, thereby promoting their elimination. Through this process, senescent cells can be recognized and removed, preventing their transformation into tumor cells. Inhibiting oncogene activity. During cellular senescence, the activity of oncogenes is suppressed. For instance, key tumor suppressors like p53 and p16 are activated during senescence, thereby preventing malignant transformation of cells (267). Senescent cells play a significant role in maintaining tissue homeostasis and preventing carcinogenesis (272). By restricting excessive cell proliferation and promoting orderly apoptosis, senescence mechanisms ensure normal tissue function and structural integrity (273). Even though cellular senescence is associated with certain pro-inflammatory symptoms and potential negative effects, its positive role in preventing malignant tumor progression is undeniable (274). By promoting cell cycle arrest and activating immune surveillance, cellular senescence plays a critical role in cancer prevention and treatment (275). Therefore, understanding how to enhance the anti-cancer functions of cellular senescence is an important focus in cancer research.

## mTOR kinase in nutrient sensing and metabolic regulation

The mTOR kinase is involved in sensing high amino acid concentrations and regulating various aspects of anabolic metabolism (276). In cancer, mTOR is often activated, stimulating cancer cell growth, promoting adaptive evolution, and driving tumor metabolic reprogramming through the re-regulation of glucose, amino acids, nucleotides, fatty acids, and lipid metabolism (277). Various mTOR-targeted drugs have been developed, including different rapalogs, to interfere with cancer cell metabolism; these drugs are also explored as part of anti-aging strategies. In contrast, the roles of AMPK and sirtuins are associated with IIS and mTOR antagonism, as they stimulate catabolic rather than anabolic metabolism in response to nutrient scarcity (198). Consequently, AMPK activation can phosphorylate and disrupt key pathways involved in cell growth and cancer cell stemness, making it a potential target for anti-cancer drugs. SIRT2 enhances the dimerization of BARD1 with the tumor suppressor BRCA1 by deacetylating BARD1, thus improving BRCA1-dependent DNA double-strand break repair (278).

## Metabolic reprogramming in cancer and aging

Cancer cells typically redirect their bioenergetics toward glycolysis and glutaminolysis (279). Overall, these changes associated with anabolic signaling promote aging and cancer, whereas reduced nutrient-sensing signals contribute to lifespan extension and decreased cancer progression, possibly due to reduced proliferation and metabolic rates mitigating cellular damage (280). In this regard, drug treatments that simulate nutrient deprivation and various dietary interventions based on continuous caloric restriction, intermittent fasting, ketogenic diets, or specific nutrient reductions (e.g., methionine) have shown promising results (281). However, designing more effective anti-aging and anti-cancer nutritional interventions requires further understanding of the molecular mechanisms underlying cancer and aging-related metabolic changes. It is important to note that metabolic characteristics of individual cancer subtypes are highly diverse, and influenced by specific oncogenes and pathways.

## Immune evasion in senescence-driven cancer

For cancer to progress to a clinically detectable stage, it must evade immune surveillance, which implies that cancer cells either “hide” from immune recognition or actively suppress immune effector cells (282). Over the past decade, this aspect of cancer has gained increasing importance, with immune checkpoint inhibitors targeting PD-1 and PD-L1 interactions, becoming pivotal in cancer treatment (283). However, certain features of senescence may contribute to cancer immune evasion, the gradual depletion of spermidine in aging tissues leads to autophagy failure, which is expected to impact the immune recognition of cancer cells. Oral spermidine supplementation has been shown to restore immune control over cancer in mouse models, and adding spermidine to culture media can restore immune responses in human B and T cells from elderly donors *in vitro (* 284). Senescent cells are often cleared by macrophages and NK cells, indicating selective immune evasion pressure. This accumulation physically obstructs T lymphocyte recognition of malignant cells, creating an immune desert within the tumor (285). Understanding these mechanisms of immune evasion in senescence-driven cancer is essential for developing strategies to overcome immune resistance and enhance the efficacy of cancer immunotherapies.

## The limitations and challenges of targeting m6A modifications in senescence therapy

While targeting m6A modifications holds great potential for senescence therapy, several limitations and challenges must be addressed before its clinical translation. First, the dynamic and reversible nature of m6A modifications, mediated by “writers” (methyltransferases), “erasers” (demethylases), and “readers” (binding proteins), adds significant complexity to their regulation. Targeting these components may lead to off-target effects due to the widespread role of m6A in various cellular processes beyond senescence, such as stem cell maintenance, immune responses, and tumor progression. This non-specificity could result in unintended disruptions to normal physiological functions. Furthermore, the heterogeneity in senescence mechanisms across cell types and tissues complicates the development of universal m6A-targeted therapies. A therapy effective in one cellular context may exhibit limited efficacy or even adverse effects in another.

Finally, the detection and quantification of m6A modifications remain challenging due to the lack of standardized and highly sensitive tools. Current techniques, such as m6A-seq, provide valuable insights but have limitations in resolution and scalability. Without precise detection methods, identifying specific m6A targets for therapeutic intervention is difficult, which hinders progress in the field. Developing small molecules or RNA-based therapeutics targeting m6A modulators poses challenges related to specificity and toxicity. Ensuring efficient delivery to senescent cells while avoiding off-target effects in non-senescent cells remains a major hurdle. Advanced delivery systems, such as nanoparticle-based or cell-specific delivery platforms, are needed but require further optimization for clinical use. Overcoming these limitations requires a multidisciplinary approach, integrating advances in molecular biology, bioinformatics, drug delivery systems, and clinical research. Continued efforts to refine m6A-targeted strategies and deepen our understanding of m6A’s role in senescence will pave the way for safer and more effective therapies.

## The limitations and challenges of cellular senescence biomarkers

Senescent cells exhibit significant heterogeneity across different tissues, cell types, and stress stimuli. This variability complicates the identification of universal biomarkers, as specific markers may only reflect certain senescence subtypes or contexts. For instance, biomarkers like p16INK4a and SA-β-gal are not uniformly expressed in all senescent cells, limiting their reliability and generalizability. Senescence is a dynamic process characterized by transitions in gene expression and phenotypic changes over time. Biomarkers may vary at different stages of senescence, making it challenging to identify markers that consistently represent the entire process. This temporal variability reduces the utility of static biomarkers for longitudinal studies or clinical monitoring.

Many proposed senescence biomarkers, such as SASP components (e.g., IL-6, IL-8) or cell cycle inhibitors (e.g., p21), are also associated with other cellular states, such as inflammation, quiescence, or differentiation. This overlap complicates the distinction between senescence and other physiological or pathological conditions, reducing diagnostic specificity. Current methods for detecting senescence biomarkers, including histochemical staining, immunohistochemistry, and transcriptomic analysis, face technical challenges. For example, SA-β-gal staining is prone to false positives under certain conditions, and transcriptomic approaches may not capture post-transcriptional or post-translational modifications. Additionally, these methods are often labor-intensive, require high expertise, or lack scalability for high-throughput applications. Many senescence biomarkers have been identified and validated in preclinical models but lack robust validation in human clinical samples. Factors such as individual variability, comorbidities, and differences in tissue microenvironments complicate the extrapolation of findings to clinical settings. Without reliable validation, the translation of senescence biomarkers into clinical diagnostics or therapeutic monitoring remains limited.

## Discussion

Cellular senescence serves as a critical biological process with both beneficial and detrimental implications, depending on the context. On one hand, senescence is essential for tissue repair, embryogenesis, and tumor suppression by halting the proliferation of damaged cells (286). On the other hand, the accumulation of senescent cells and their senescence-associated secretory phenotype (SASP) contribute to chronic inflammation, tissue dysfunction, and the progression of age-related diseases such as cancer, diabetes, and neurodegenerative disorders (287). This duality highlights the complexity of senescence and underscores the need for precise modulation to harness its positive effects while mitigating its harmful outcomes.

Epigenetic modifications, particularly m6A RNA methylation, have emerged as pivotal regulators of cellular senescence. The dynamic interplay between m6A writers, erasers, and readers modulates gene expression and cellular responses under various stress conditions. For instance, m6A-seq studies have revealed critical regulatory pathways, such as p53 and NF-κB signaling, that are influenced by m6A modifications during senescence (241, 245). Despite these advancements, several challenges remain. The specificity of m6A modulators and their downstream effects require deeper investigation, and the lack of standardized tools for detecting and quantifying m6A modifications limits their translational potential.

In the therapeutic realm, senescence-targeting strategies, including senolytics and senomorphics, show great promise but face significant hurdles. While senolytics effectively eliminate senescent cells, their off-target effects and potential toxicity limit clinical applicability. Senomorphics, which modulate SASP, offer a less invasive approach but require precise delivery systems to avoid unintended consequences. Additionally, the identification of robust and specific biomarkers for senescence remains an urgent priority to enable accurate diagnosis and monitoring of therapeutic outcomes. Addressing these challenges through interdisciplinary research and innovative technologies will be critical to advancing our understanding and utilization of cellular senescence in disease prevention and treatment.

## Conclusions

Cellular senescence plays a dual role in maintaining physiological homeostasis and contributing to pathological processes such as aging and cancer. Understanding the intricate mechanisms underlying senescence, particularly the regulation by epigenetic modifications like m6A, provides valuable insights into its functional complexity. Advances in anti-senescence therapies and biomarkers hold promise for mitigating age-related diseases and enhancing cancer treatment. However, challenges such as off-target effects, therapy resistance, and limited clinical translation underscore the need for further research. A comprehensive approach integrating molecular insights with innovative therapeutic strategies could pave the way for transformative advancements in targeting cellular senescence.
